# Upper respiratory tract immunization with Pam_2_Cys-adjuvanted spike protein vaccine achieves sterilizing protection against SARS-CoV-2

**DOI:** 10.3389/fimmu.2025.1654126

**Published:** 2025-09-02

**Authors:** Erica L. Stewart, Skye Stockdale, Matt D. Johansen, Lachlan Smith, Sibel Alca, Joshua W. C. Maxwell, Duc H. Nguyen, Stefan Miemczyk, Guanshu Zhao, Stuart Turville, James A. Triccas, Megan Steain, Scott N. Byrne, Philip M. Hansbro, Richard J. Payne, Warwick J. Britton, Anneliese S. Ashhurst

**Affiliations:** ^1^ Tuberculosis Research Program, Centenary Institute, The University of Sydney, Sydney, NSW, Australia; ^2^ Sydney Institute for Infectious Diseases and the Charles Perkins Centre, The University of Sydney, Sydney, NSW, Australia; ^3^ School of Medical Sciences, Faculty of Medicine and Health, The University of Sydney, Sydney, NSW, Australia; ^4^ Centre for Inflammation, Centenary Institute and University of Technology Sydney, Faculty of Science, School of Life Sciences, Sydney, NSW, Australia; ^5^ School of Chemistry, Faculty of Science, The University of Sydney, NSW, Australia; ^6^ Australian Research Council Centre of Excellence for Innovations in Peptide and Protein Science, The University of Sydney, Sydney, NSW, Australia; ^7^ The Kirby Institute, University of New South Wales, Sydney, NSW, Australia; ^8^ Department of Clinical Immunology, Royal Prince Alfred Hospital, Sydney, NSW, Australia

**Keywords:** nasal vaccines, mucosal immunity, subunit vaccination, mucosal adjuvant, COVID-19, SARS-CoV-2, mucosal vaccine, TLR2 agonist

## Abstract

Injected COVID-19 vaccines protect against severe disease, but do not induce robust mucosal immune responses. Nasal vaccines offer the advantage of local immunity to block viral infection and transmission. Previously we showed immunization of a Pam_2_Cys-adjuvanted SARS-CoV-2 vaccine to the upper and lower respiratory tracts (URT/LRT) induced protective immune responses in the lungs. However, URT/LRT immunization is not representative of nasal vaccines for clinical use that exclusively target the URT. Here, we show that delivery to only the URT with Pam_2_Cys and spike protein effectively induced strong SARS-CoV-2 specific immune responses in the nasal mucosa. When delivered in a low volume so that vaccine exposure was limited to the URT, Pam_2_Cys/spike protein induced local SARS-CoV-2-specific Th17 cells and neutralizing antibodies to a similar level to inhaled vaccination reaching both the URT and LRT. We compared URT versus URT/LRT delivery as booster vaccinations following parenteral immunization and found that URT vaccination concentrated the immune response to the URT rather than the lungs. Importantly, URT immunization or boosting induced sterilizing immunity in K18-hACE2 mice challenged with homologous SARS-CoV-2. Thus, booster vaccination to the URT alone with Pam_2_Cys/spike achieved robust nasal immunity against SARS-CoV-2 and is a promising strategy for clinical development.

## Introduction

Current severe acute respiratory coronavirus-2 (SARS-CoV-2) vaccines, including those updated for recent Omicron variants, are adept at reducing disease severity but provide only partial protection against initial SARS-CoV-2 infection and ongoing transmission (1). Moreover, continued circulation of SARS-CoV-2 in vaccinated populations fuels the emergence of vaccine-resistant variants. Animal studies demonstrate that mucosal delivery of vaccines can generate immune responses in the respiratory tract that increase protection against infection and reduce viral transmission (2–4). Furthermore, nasal vaccines are attractive candidates because of ease of delivery and increased vaccine acceptability (5). Despite these advantages, few nasal vaccines are approved for clinical use. Live-attenuated intranasal (IN) vaccines for influenza were first used clinically in 1987 (6) and demonstrated to generate mucosal IgA and provide significant protection against infection (7). Since the coronavirus of 2019 (COVID-19) pandemic, five more inhaled vaccines have been approved for clinical use (4, 8). While there are several IN vaccines undergoing clinical trials, the majority are viral vectored or live-attenuated vaccines with a scarcity of subunit vaccines. This is mostly owing to a lack of vaccine adjuvants suitable for IN delivery, either for safety reasons or incompatibility with the mucosal immune environment.

The vaccine adjuvant dipalmitoyl-*S*-glycerylcysteine (Pam_2_Cys) is a lipopeptide that activates toll-like receptor (TLR)2/6 on immune and epithelial cells and is an effective mucosal adjuvant in preclinical mouse models (9, 10). When fused to a *Mycobacterium tuberculosis* secreted protein and delivered to the lungs of mice, Pam_2_Cys promoted a local Th17 phenotype and protection against *M. tuberculosis* challenge (11, 12). When Pam_2_Cys was delivered with the SARS-CoV-2 ancestral spike protein in a large volume by the IN route to vaccinate both the URT and LRT, this adjuvant stimulated robust spike-specific IgA and IgG alongside Th17-polarized immune memory in the lower respiratory tract (10).

Targeting the upper airways alone for vaccination reduces the risk of damage to the lower airways and lungs. Furthermore, to prevent SARS-CoV-2 infection of epithelial cells in the upper airways, immune memory in the URT is crucial. In the current study, we used a validated method of URT delivery of Pam_2_Cys/spike to examine the capacity for this vaccine to generate lasting SARS-CoV-2-specific immune responses in the nasal passages – the initial site of SARS-CoV-2 infection. We compared URT-only delivery to URT/LRT delivery for the ability to generate mucosal and systemic immune responses and protect against infection with SARS-CoV-2 (9).

## Results

### URT vaccination with Pam_2_Cys plus SARS-CoV-2 spike protein induces comparable immune responses in the respiratory tract to URT/LRT vaccination

**Figure 1:**
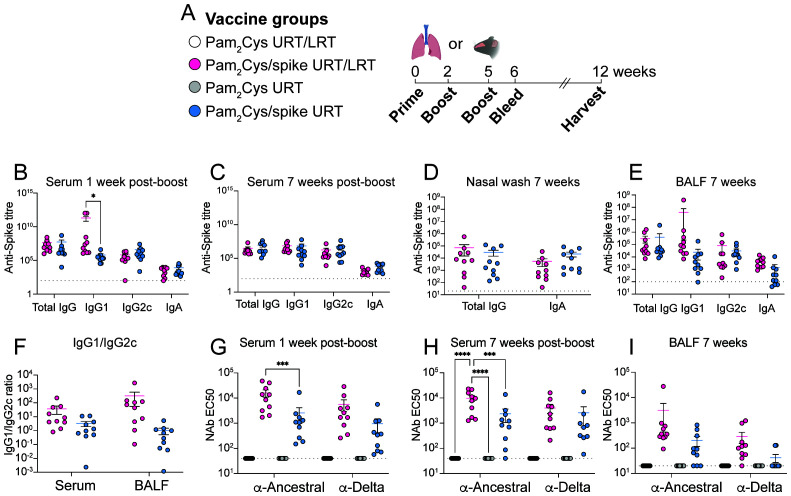
URT vaccination generated comparable humoral immune responses to URT/LRT delivery in both the circulation and respiratory tract. C57BL/6 mice were immunized with 5 µg Pam_2_Cys and 6 µg ancestral spike protein via the upper respiratory tract (URT) or upper and lower respiratory tract (URT/LRT) delivery as per the schedule shown in **(A)**. Serum was collected one week after the final boost and blood and other tissues (BALF and nasal wash) were harvested after 7 weeks. Serum was analyzed at each time point **(B, C)**, and nasal wash and BALF were analyzed at 7 weeks **(D, E)** for anti-spike antibody titers using ELISA. Samples from adjuvant-only groups were pooled and the average plus three standard deviations of the absorbance were used as the negative control cutoff point to determine antibody titers. Ratio of total IgG1/IgG2c in the serum and BALF at 7 weeks post-boost are shown in **(F)**. Serum **(G, H)** and BALF **(I)** were also analyzed for neutralizing antibodies (NAb) using pseudovirus assay. Data are pooled from two independent experiments with a total of n=10 per group showing mean +/- SEM. **(B-F)** were analyzed for differences using a two-tailed Mann-Whitney test, **(G-I)** were analyzed using a 2-way ANOVA with Tukey *post-hoc* test. For all graphs *p* < 0.0332(*), *p* < 0.0021(**), *p* < 0.0002 (***), *p* < 0.0001(****), and dotted lines depict limit of detection.

Current COVID-19 vaccines are highly effective at reducing disease severity, but a major aim of next-generation SARS-CoV-2 vaccines is to generate immune memory in the upper airways that may prevent infection and transmission. In a previous study, we showed that Pam_2_Cys combined with ancestral spike protein induced robust spike-specific antibody and T cell responses after nasal administration to both the URT and LRT of mice (30-50 µL total volume) (10). However, nasal vaccine administration to the nose and lung is not reflective of nasal vaccines used in clinical settings that only target the upper airways, and immune responses to URT immunization with Pam_2_Cys/spike vaccine has not been assessed. Therefore, we used low-volume nasal administration (6 µL per nare) that restricts vaccine delivery to the nasal passages (9) (hereafter termed URT administration). Animals were immunized three times (one prime, two boost) then rested for seven weeks before assessment of memory immune responses in the upper and lower respiratory tracts and in the blood (**Figure 1A**, **Supplementary Figure S1A**). URT vaccination of mice with ancestral HexaPro spike protein (6 µg) and Pam_2_Cys adjuvant (5 µg) formulated in phosphate-buffered saline (PBS) induced spike-specific antibodies, including IgA, in the blood, the nasal passages (nasal wash) and lungs (bronchoalveolar fluid; BALF) comparable to traditional URT/LRT delivery (**Figures 1B-F**). No significant differences were observed between URT and URT/LRT delivery, with the exception that URT/LRT delivery initially induced higher IgG1 titers in the serum one week after immunization, but this reached the same levels as URT delivery at the memory timepoint of 7 weeks (**Figures 1B, C**). Neutralizing antibodies (NAb) are a correlate of vaccine-induced protection against SARS-CoV-2 (13). URT vaccination induced high levels of NAb against homologous pseudovirus in both the blood and respiratory tract, although these were lower than following URT/LRT vaccination (**Figures 1G-I**). URT-only delivery, however, induced comparable cross-neutralizing antibodies against pseudovirus expressing the delta variant spike protein, as compared with URT/LRT delivery. The presence of NAb were also assessed in the nasal wash, but none of the vaccination strategies tested induced significant NAb in these samples (**Supplementary Figure S1B**). Thus, URT vaccination with Pam2Cys/spike generated circulating spike-specific and neutralizing antibodies in the blood and airways.

**Figure 2:**
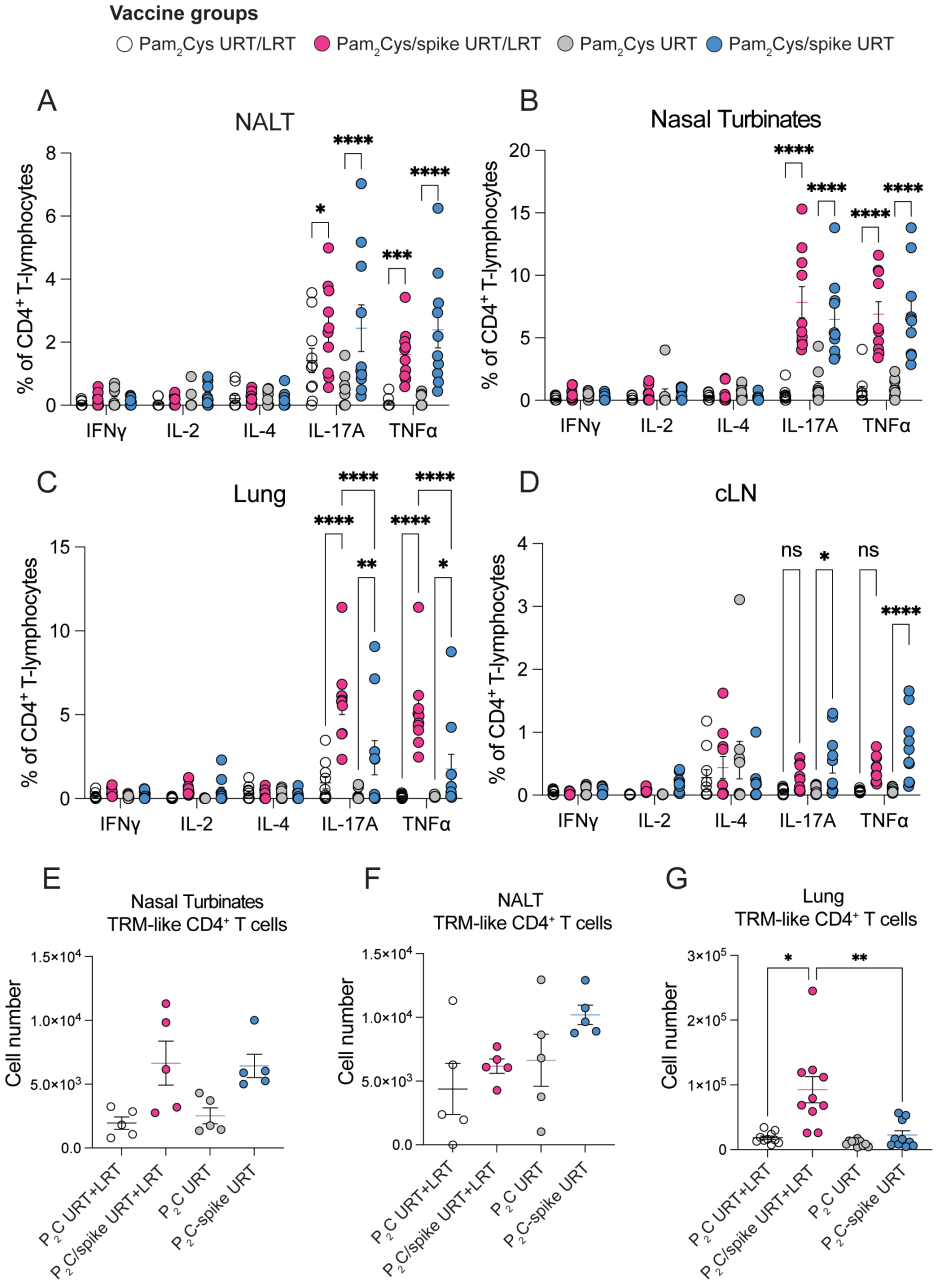
URT vaccination promoted the retention of TRMs in the nasal turbinates and NALT. C57BL/6 mice were immunized with 5 µg Pam_2_Cys and 6 µg ancestral spike protein either via the upper respiratory tract (URT) or upper- and lower-respiratory tract (URT/LRT). Mice were immunized as per the schedule shown in **Figure 1A**, with tissues harvested at 7 weeks post-boost. Antigen-specific T cells were recalled from the NALT, nasal turbinates, lungs and cervical lymph node (cLN) of mice **(A-D)** and intracellular cytokine expression was detected by antibody labelling and flow cytometry. Tissue-resident memory T cells (TRM-like) were defined as CD4^+^CD69^+^CD44^+^CD62L^-^ or CD8^+^CD69^+^CD44^+^CD103^+^CD62L^-^ in the lungs, nasal turbinates and NALT **(E-G)**. For **(A-D, G)** data is pooled from two independent experiments with a total of n=10 per group and data was analyzed for differences using a 2-way ANOVA with Tukey *post-hoc* test. For **(E, F)**, data is representative of a single experiment with n=5 per group. Differences were calculated using a Kruskal-Wallis test with Dunn’s *post-hoc* test. All graphs show mean +/- SEM and *p* < 0.0332(*), *p* < 0.0021(**), *p* < 0.0002 (***), *p* < 0.0001(****).

Tissue-resident memory T cells (TRM) are associated with increased protection against respiratory pathogens and cross-protection against different SARS-CoV-2 variants (14). After URT/LRT administration, Pam_2_Cys induces a Th17 phenotype of T cell responses in the lungs (10). In this study, we examined the nasal-associated lymphoid tissue (NALT), which is the murine equivalent of the Waldeyer’s ring in humans, and the nasal turbinates for vaccine-specific T cell responses to Pam_2_Cys/spike. URT Pam_2_Cys/spike induced significant levels of spike-specific CD4^+^ T cells expressing IL-17A and TNF in the nasal passages, lungs and local draining lymph node compared with Pam_2_Cys adjuvant-only controls (**Figures 2A-D**). However, CD4^+^ T cell responses were significantly reduced in the lungs of URT-only immunized mice compared to URT/LRT immunization (**Figure 2C**). In accordance with the previous study (10), low levels of antigen-specific CD8^+^ T cell responses were detected in the lungs and NALT after URT/LRT immunization, but were not detected in the nasal turbinates or cervical lymph node (cLN) (**Supplementary Figures S1C-F**). Further, URT vaccination induced notable levels of TRM cells in the nasal passages (**Figures 2E, F**) with reduced levels in the lungs (**Figure 2G**) compared to URT/LRT administration.

**Figure 3:**
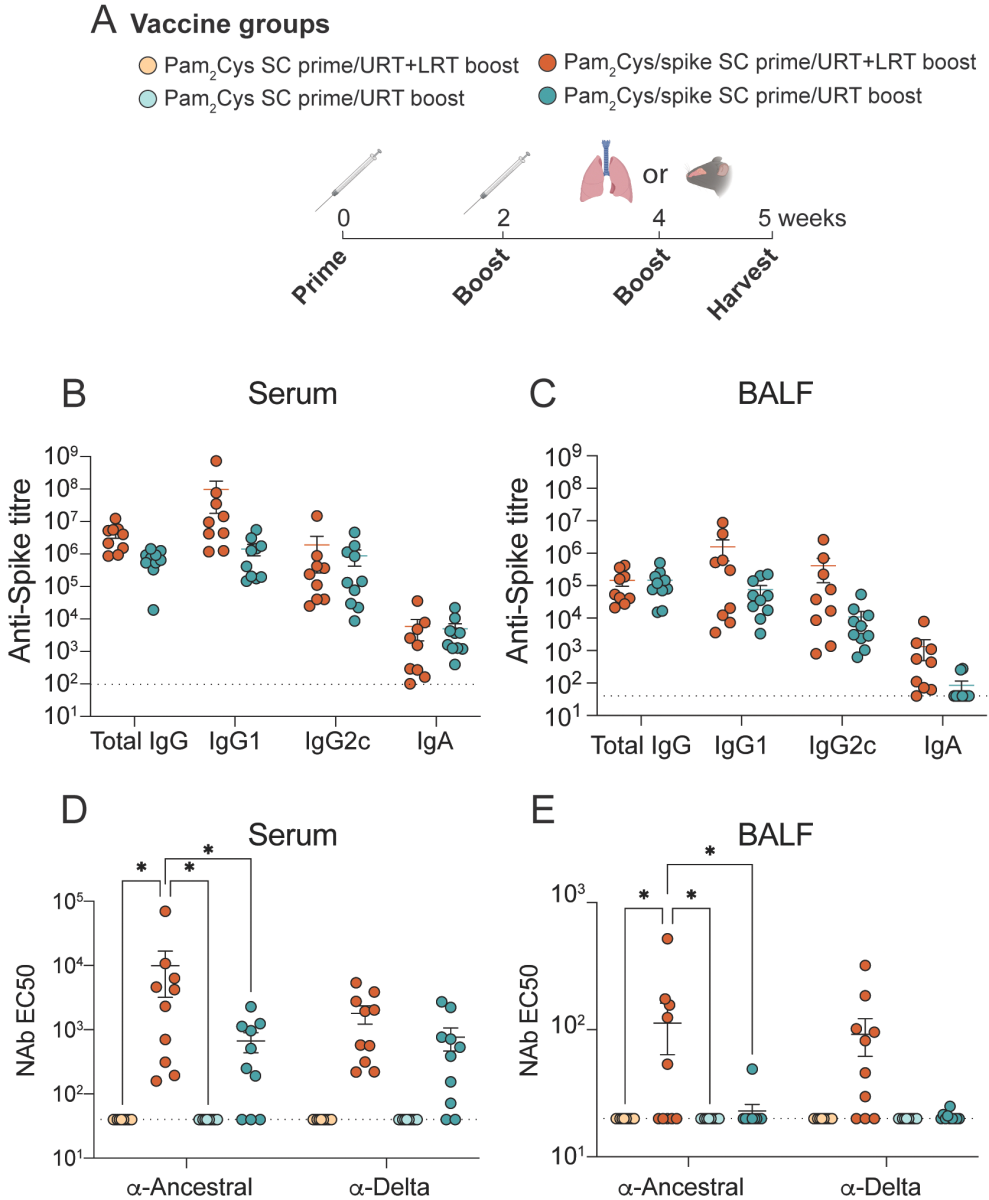
Mucosal boosting with URT vaccination generated anti-spike humoral immune responses in the circulation and respiratory tract. C57BL/6 mice were immunized with 5 µg Pam_2_Cys and 6 µg ancestral spike protein twice subcutaneously (SC) followed by upper respiratory tract (URT) or upper- and lower-respiratory tract (URT/LRT) delivery as outlined in **(A)**. One-week after the final vaccination, serum and BALF were analyzed for the presence of anti-spike antibodies **(B, C)** or neutralizing antibodies (NAb) using pseudovirus assay **(D, E)**. Samples from adjuvant-only groups were pooled and the average plus three standard deviations of the absorbance were used as the negative control cutoff point to determine antibody titers. Data were pooled from two independent experiments with a total of n=10 per group. **(B, C)** were analyzed for differences using a two-tailed Mann-Whitney test, data in **(D, E)** were analyzed using a 2-way ANOVA with Tukey *post-hoc* test. For all graphs *p* < 0.0332(*) and dotted lines depict limit of detection.

Because of the widespread use of injectable COVID vaccines, mucosal vaccines receiving future approval for clinical use are likely be used as booster vaccines following prior parenteral vaccination. Thus, we next tested Pam_2_Cys/spike URT as a mucosal booster vaccine after injected (subcutaneous; SC) prime immunization (**Figure 3A**, **Supplementary Figure S1G**). URT boosting with Pam_2_Cys/spike induced similar titers of anti-spike antibodies in the serum, BALF and nasal wash one-week post final vaccination compared to boosting with a URT/LRT vaccination, with a trend towards reduced titers in the BALF (**Figures 3B, C**; **Supplementary Figure S1H**). URT immunization induced NAb against both ancestral and delta-variant pseudoviruses in the serum, although URT/LRT immunization induced greater titers in the BALF and serum (**Figures 3D, E**). Neither booster strategy induced significant NAb titers in the nasal wash (**Supplementary Figure S1I**).

**Figure 4:**
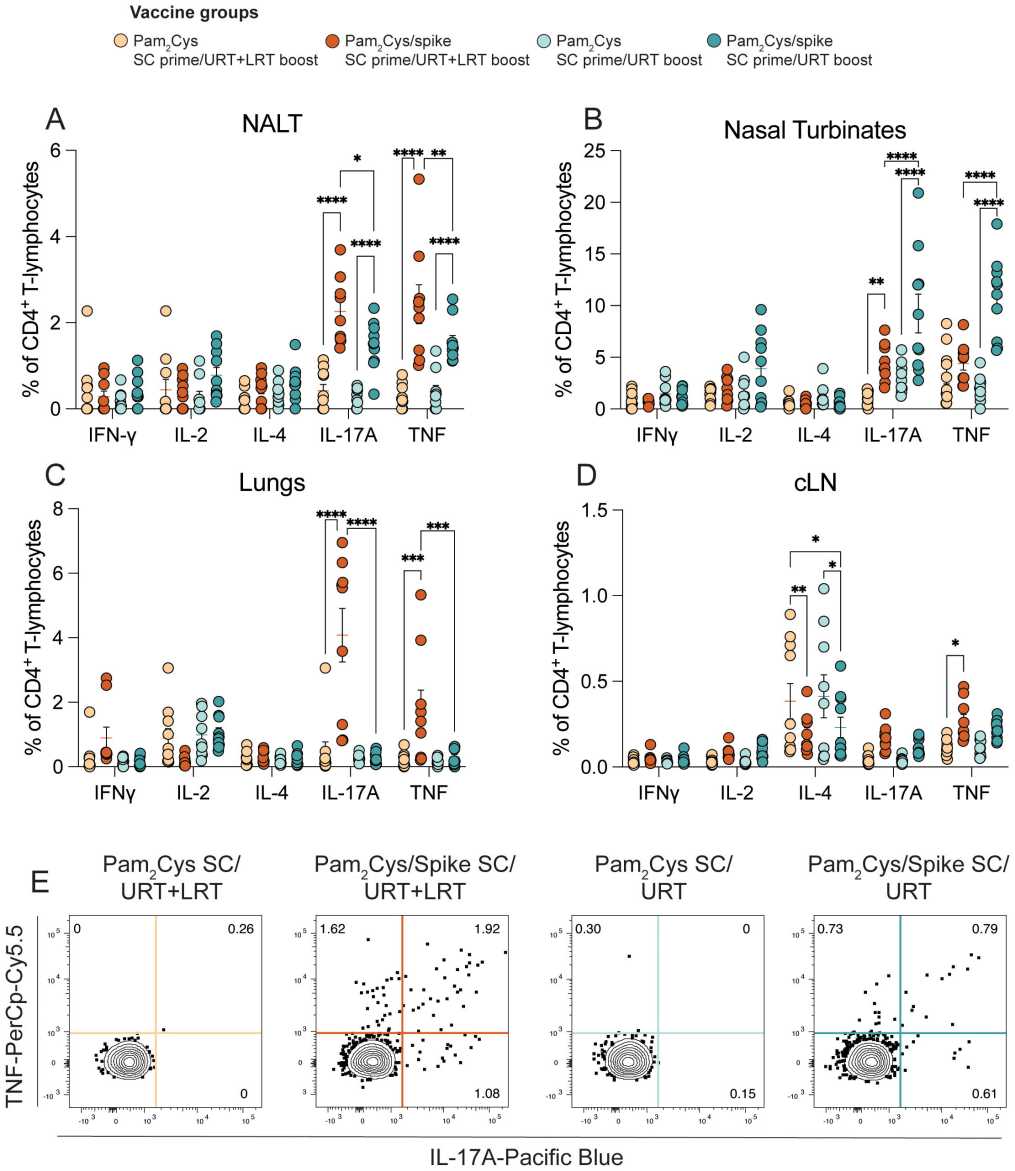
URT booster vaccination generated robust antigen-specific CD4^+^ T cell responses in the upper respiratory tract. C57BL/6 mice were immunized with 5 µg Pam_2_Cys and 6 µg ancestral spike protein twice subcutaneously followed by upper respiratory tract (URT) or upper- and lower-respiratory tract (URT+LRT) boosting as shown in **Figure 3A**. One week later, single cell suspensions of the NALT, nasal turbinates, lungs and cLN **(A-D)** were restimulated with spike protein and examined using intracellular cytokine staining. Representative dot plots of CD4^+^ T cells in the NALT expressing IL-17A and TNF are shown in **(E)**. Data are pooled from two independent experiments of a total of n=10 mice per group showing mean +/- SEM. Differences between groups were analyzed using a 2-way ANOVA with *post-hoc* Tukey test. For all graphs *p* < 0.0332(*), *p* < 0.0021(**), *p* < 0.0002 (***), *p* < 0.0001(****).

We also compared cellular responses after URT versus URT/LRT boosting. URT boosting of Pam_2_Cys/spike induced increased antigen-specific Th17 cytokine responses in the nasal turbinates compared to URT/LRT boosting, which favored greater responses in the NALT and lungs (**Figures 4A-C, E**). Only URT/LRT delivery induced a small but significant increase in TNF expression in CD4^+^ T cells in the cervical lymph node (cLN; **Figure 4D**). Similarly to when three mucosal vaccinations were administered, URT/LRT boosting induced low levels of antigen-specific CD8^+^ T cells in the lungs and nasal turbinates (**Supplementary Figures S1J-M**). Thus, URT immunization stimulates URT humoral and CD4^+^ T-cell immunity when used as a mucosal booster to parenteral vaccination, albeit at lower levels than URT/LRT boosting.

### Pam_2_Cys and D-spike enhances URT immune responses compared to protein alone

**Figure 5:**
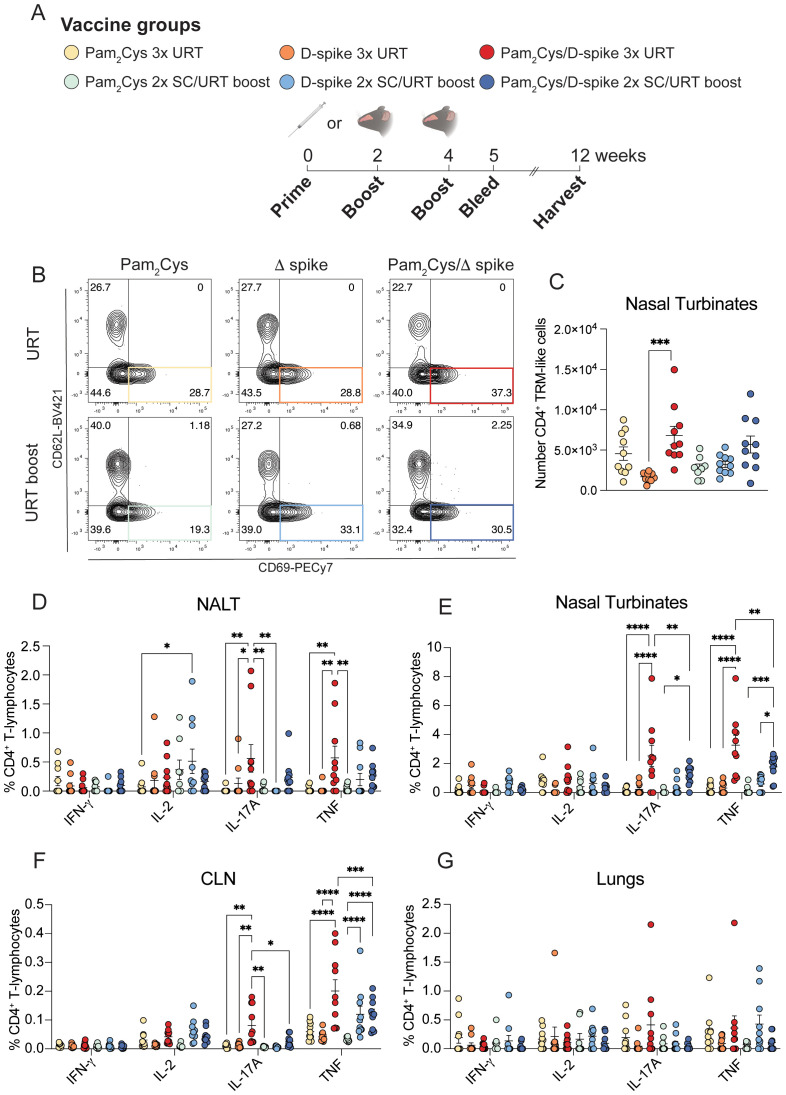
URT immunization with D-spike vaccine generated T cell responses in the upper respiratory tract. C57BL/6 mice were immunized with 5 µg Pam_2_Cys and 6 µg delta variant spike (D-spike) protein either twice subcutaneously followed by upper respiratory tract (URT) boosting or a full schedule of URT delivery as shown in **(A)**. Representative FACS plots of TRM-like CD4^+^ T cells (defined as CD4^+^CD44^+^CD69^+^CD62L^-^) in the nasal turbinates **(B)**, with total number of cells **(C)**. Single cell suspensions were restimulated with D-spike protein and the flow cytometric analysis of intracellular cytokine staining of CD4^+^ T cells from the NALT, nasal turbinates, cLN and lungs are shown **(D-G)**. Data are pooled from two independent experiments with a total of n=9–10 per group with mean +/- SEM. Differences were calculated in **(B)** using a 1-way ANOVA with *post-hoc* Tukey test. Differences in **(D-G)** were calculated using 2-way ANOVA with *post-hoc* Tukey test. For all graphs *p* < 0.0332(*), *p* < 0.0021(**), *p* < 0.0002 (***), *p* < 0.0001(****).

Next, we examined if URT boosting of parenteral immunization is as effective at inducing local immune responses as multiple URT vaccinations in a head-to-head comparison. Using delta-variant spike protein (D-spike) as antigen, mice were immunized either twice SC or twice URT, followed by a single URT booster (**Figure 5A**, **Supplementary Figure S2A**) with D-spike protein alone, D-spike with Pam_2_Cys or Pam_2_Cys alone. Three URT immunizations induced significant levels of TRM-like CD4^+^ T cells in the nasal turbinates, defined as CD44^+^CD69^+^CD62L^-^ CD4^+^ T cells, while URT boosting resulted in a trend for an increase in these cells (**Figures 5B, C**). When examining antigen-specific CD4^+^ T cell responses, three URT immunizations induced significant IL-17A and TNF expression in the NALT, while URT boosting did not (**Figure 5D**). Both strategies, however, induced significant cytokine expression in the nasal turbinates and nasal-draining lymph node (**Figures 5E, F**). Neither protocol of URT immunization stimulated significant cytokine-expressing T cells in the lungs, however there was a slight trend towards IL-17A expression after three URT immunizations (**Figure 5G**). No significant CD8^+^ T cell responses were observed in any of the tissues examined (**Supplementary Figures S2B-E**).

**Figure 6:**
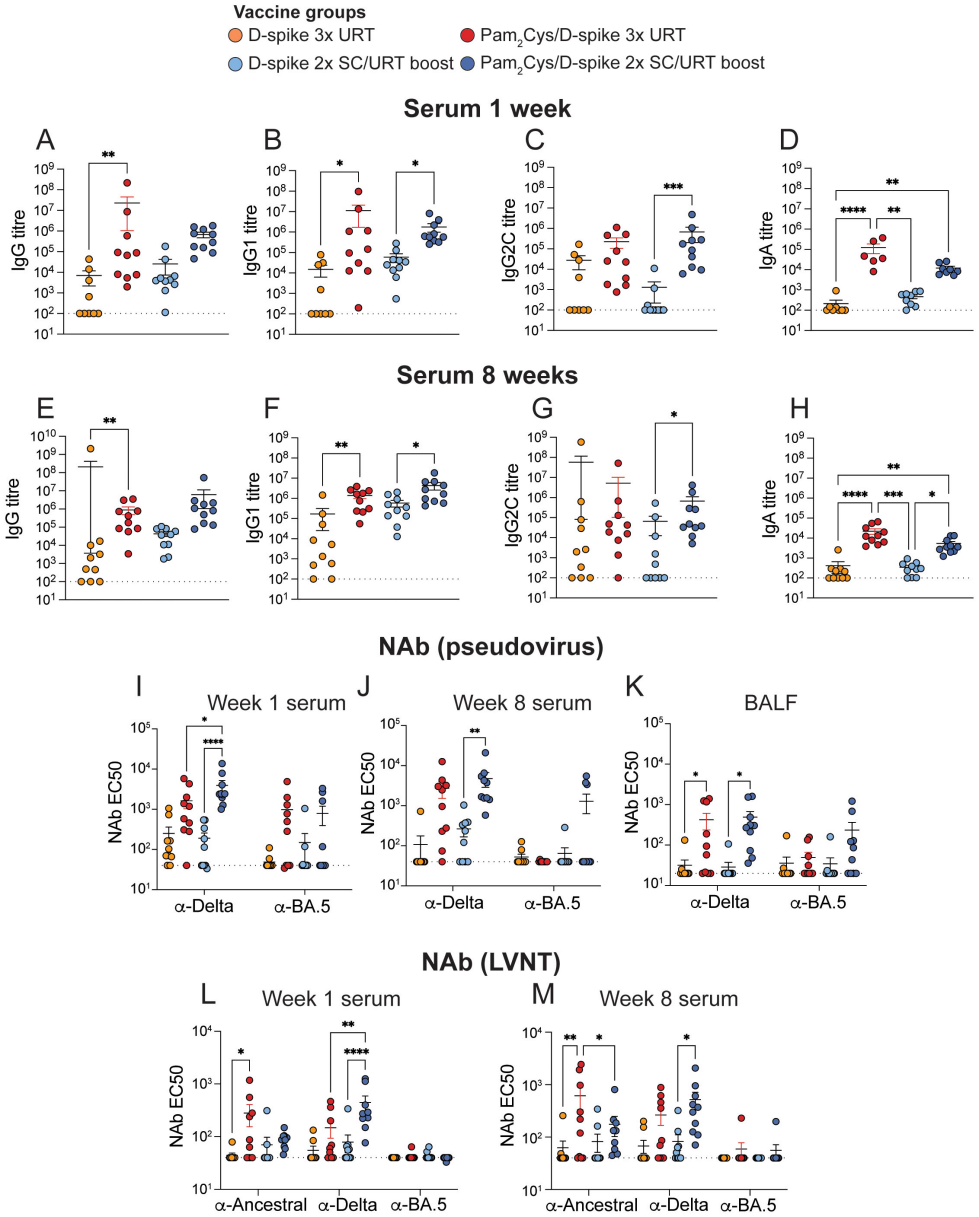
URT delivery of Pam_2_Cys/spike promoted local and circulating spike-specific antibodies and NAb. C57BL/6 mice were immunized with 5 µg Pam_2_Cys and 6 µg delta variant spike (D-spike) protein either twice subcutaneously followed by an upper respiratory tract (URT) booster or a full schedule of URT delivery as shown in **Figure 5A**. Levels of anti-spike IgG, IgG1, IgG2c, and IgA were examined in the serum at one- **(A-D)** and 8-weeks post-immunization **(E-H)**. Samples from adjuvant-only controls were pooled and used to determine the cutoff value for antibody titers. Neutralizing antibodies (NAb) were assessed in the serum (week 1 and 8) and BALF (week 8 only) using pseudovirus **(I-K)** and live virus **(L, M)** neutralization assays. Differences in **(A-H)** were analyzed using 1-way ANOVA with *post-hoc* Dunn’s test and **(I-M)** were analyzed using 2-way ANOVA with *post-hoc* Tukey test. Data is pooled from two independent experiments with n=9–10 per group and depicts mean +/- SEM, where *p* < 0.0332(*), *p* < 0.0021(**), *p* < 0.0002(***), *p* < 0.0001(****). Dotted lines indicate limit of detection of assay.

In the humoral compartment, URT boosting promoted high levels of D-spike-specific antibodies in the serum at 1- and 8-weeks post-immunization (**Figures 6A-H**, **Supplementary Figures S2F, G**). Furthermore, URT boosting with Pam_2_Cys/D-spike induced high NAb titers in the serum at both timepoints post-immunization, as measured by both pseudovirus and live virus neutralization assays (**Figures 6I, J, L, M**). URT immunization induced significant spike-binding and NAb in the BALF against delta pseudovirus, but no significant responses were measured in the nasal wash (**Figure 6K**; **Supplementary Figures S2H-K**). Overall, these data demonstrate that URT boosting with D-spike protein adjuvanted with Pam_2_Cys adjuvant generates robust cellular and humoral immune responses against SARS-CoV-2 in the URT of mice.

### URT vaccination with Pam_2_Cys and delta-variant spike protein alone or as booster protects against SARS-CoV-2 infection

**Figure 7:**
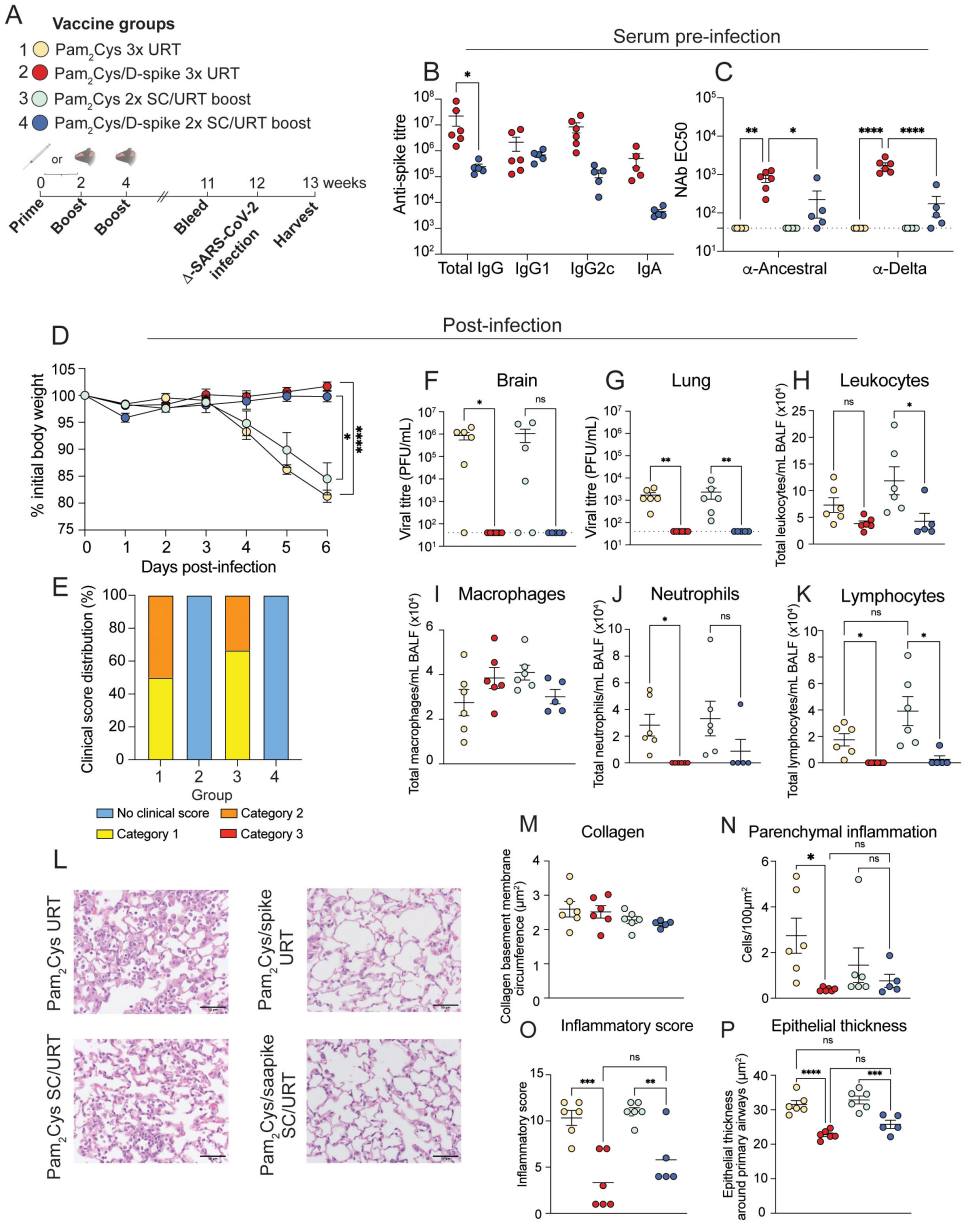
URT immunization with D-spike vaccine provided sterilizing protection against homologous SARS-CoV-2 challenge. K18-hACE2 mice were immunized with Pam_2_Cys and D-spike protein either twice subcutaneously (SC) followed by upper respiratory tract (URT) boosting or a full schedule of URT delivery as shown in **(A)**. SC vaccinated mice were first immunized with 5 µg Pam_2_Cys and 6 µg D-spike protein, followed by a booster with 3 µg Pam_2_Cys and 6 µg D-spike protein. All nose-only vaccinations used 5 µg Pam_2_Cys/6 µg D-spike protein. Eight weeks after immunization mice were challenged intranasally with 10^3^ PFU of delta-variant SARS-CoV-2. Anti-spike antibody titers and live virus NAb titers against ancestral and delta SARS-CoV-2 were assessed prior to challenge **(B, C)**. Body weight after challenge is shown in **(D)**, and clinical scoring **(E)** was also performed. Viral titers of the brain and lung collected 6 days post-infection are depicted in **(F, G)**. Infiltration of leukocytes, macrophages, neutrophils and lymphocytes in the BALF were enumerated after challenge **(H-K)**. Histology was also performed on the lungs of infected mice **(L-P)**. Representative images are shown in **(L)** with scoring of each animal shown in **(M-P)**. Scale bars indicate 50 µm. Data is representative of a single experiment with n=6 mice per group, showing mean +/- SEM. Differences in **(B, C)** were examined using a 2-way ANOVA with *post-hoc* Sidak and Tukey test respectively. Difference between groups in **(D)** were analyzed using 2-way ANOVA with *post-hoc* Tukey test. All other graphs were analyzed using 2-way ANOVA with *post-hoc* Dunn’s test. For all graphs, *p* < 0.0332(*), *p* < 0.0021(**), *p* < 0.0002 (***), *p* < 0.0001(****).

Finally, to test the efficacy of Pam_2_Cys/D-spike as an URT vaccine, K18-hACE2 mice (mice genetically modified to express the human cellular entry receptor for SARS-CoV-2) were immunized by the URT route with Pam_2_Cys/D-spike alone or following priming by SC immunization. The mice were then challenged 8 weeks later with 1,000 PFU delta-variant SARS-CoV-2 (**Figure 7A**). Prior to challenge, URT boosting with Pam_2_Cys/D-spike induced high levels of serum anti-spike IgG and IgA antibodies and NAb measured by live virus neutralization assay (**Figures 7B, C**). After challenge, mice immunized by either URT alone, or SC routes followed by a URT booster, were completely protected from weight loss and clinical evidence of infection (**Figures 7D, E**). Furthermore, vaccinated mice had no virus detectable in both the lungs and the brain at this timepoint (**Figures 7F, G**). The nasal turbinates were also tested for SARS-CoV-2 viral titers, but none of the animals had any viable virus in the nasal turbinates at this timepoint (data not shown). This was associated with minimal pulmonary inflammation indicated by reduced cellular infiltrate into the BALF when compared to Pam_2_Cys only controls (**Figures 7H-K**). Furthermore, URT immunization protected mice from alveolar inflammation evidenced by histological analysis (**Figures 7L-P**). Thus, either URT only mucosal immunizations with Pam_2_Cys/D-spike, or an URT boosting following peripheral priming, induced protection against SARS-CoV-2 infection characterized by undetectable viral load and completely abrogated disease in the highly susceptible K18-hACE2 mice.

## Discussion

Mucosal vaccination is a promising strategy to block initial viral infection and transmission and potentially reduce the emergence of SARS-CoV-2 variants via the generation of immune memory at the site of infection. In addition to the generation of local immune responses, IN vaccination has practical advantages over traditional vaccine delivery methods. Less invasive delivery means that some vaccine-hesitant populations can be accessed, such as children or people with fear of needles (15). In addition, recent clinical studies have shown that in some cases IN vaccines have reduced or less problematic side effects compared to their parenterally administered counterparts (16, 17). To reduce the risk of side effects and possible inflammatory damage to the lungs, nasal vaccines should primarily target the upper airways, prompting recent studies to perform low-volume nasal delivery that better reflects clinical delivery to the URT (9, 18, 19). In this study, we demonstrated that URT administration using the TLR2/6 agonist Pam_2_Cys in a low volume of vaccine delivered only to the URT provides sterilizing protection in the lungs and brain against SARS-CoV-2 in a mouse model of infection.

The URT and LRT each have distinct immune compartments, and recently it has been established that immune compartmentalization exists even within the nasal passages (20). Early innate immune responses to SARS-CoV-2 are primarily restricted to the URT, and when the virus infects the lower airways, innate responses are substantially reduced, leading to enhanced pathology and disease (21). Thus, the induction of early-responding adaptive immunity in the upper airways is pivotal to preventing SARS-CoV-2 initial infection and transmission, a feature that has thus far eluded the currently approved COVID-19 vaccines. We previously demonstrated that the adjuvanticity of Pam_2_Cys is mediated by both hematopoietic and epithelial cells after URT/LRT delivery (10). The broad equivalence observed in the magnitude of the immune responses generated by multiple URT versus URT/LRT immunizations (**Figures 1**, **2**), suggests that the adjuvant activity of intranasal Pam_2_Cys-adjuvanted vaccines can be sufficiently mediated by cells of the URT. In some cases, however, URT-only immunization resulted in lower NAb levels when compared to URT/LRT immunization (**Figure 1**), although these were sufficient for sterilizing protection in the brain and lungs of highly susceptible K18 mice at day 6 post-infection (**Figure 7**). The greater immune response induced by URT/LRT immunization may be because the lungs contain a larger surface area for vaccine to interact with epithelial and immune cells than the URT. Furthermore, pulmonary delivery engages different immune mechanisms to URT-only delivery, such as induction of inducible bronchus-associated lymphoid tissue (iBALT), a site of germinal center formation and antibody secretion (22). However, URT immunization with Pam_2_Cys/spike induced sufficient memory immune responses to provide equivalent protection to K18 mice against SARS-CoV-2 infection compared to URT/LRT delivery, supporting URT-only delivery of this Pam_2_Cys/spike vaccine.

Antigen presenting cells (APCs) in the upper airways are primarily located in the NALT in mice, corresponding to the Waldeyer’s Ring that includes the tonsils in humans (4). The upper airways are a unique immune environment that differs from the lower respiratory tract and, more recently, found to contain distinct immune compartments within this site (20, 23). Ramirez et al. demonstrated that germinal center formation occurs in the adenoid tissue of the URT in healthy adults, a tissue that was previously thought only to be a major immune site in infants and young children (20). In mice, the NALT is a site of naïve CD4^+^ T and B cell priming and memory CD8^+^ T cell recall, and nasal administration of unadjuvanted spike protein elicits a boosting effect after prior vaccination (24–26). A modified Pam_2_Cys immune modulator developed by ENA Respiratory (Australia) has recently been evaluated for prophylactic reduction of SARS-CoV-2 and influenza viral loads (9, 27). URT administration of the Pam_2_Cys analogue, INNA-X, leads to early chemokine and cytokine expression and the recruitment of neutrophils and macrophages to the upper airways, further enhancing local adaptive responses (9). Previously we demonstrated that URT/LRT delivery of Pam2Cys/spike generates robust Th17 responses in the lungs (10). Here, we show that URT vaccination with Pam_2_Cys/spike generates robust antigen-specific Th17 T cell, NAb and IgA responses in the nasal passages and circulation.

Levels of SARS-CoV-2-specific IgA correlate with increased protection from breakthrough infection with Omicron variants (28). Furthermore, secretory IgA (sIgA), present in mucosal sites, was recently shown to be the primary mechanism for neutralization of SARS-CoV-2 and protection following IN vaccination with an adenovirus-vectored vaccine, compared with serum IgA and IgG (29). We observed the induction of IgA in the nasal wash and BALF of URT-immunized mice (**Figures 1**, **3**, **5**). The induction of Th17 responses is a characteristic of multiple mucosal vaccines (30–32), but mechanisms of CD4^+^ T cell priming after nasal vaccination are underexplored. However, the induction of lung-specific TRM after mucosal, but not parenteral, delivery suggests either local CD4^+^ priming or local activation of APCs prior to migrating to the draining lymph-nodes (33, 34). Th17 cells correlate with severe disease in later stages of COVID-19, but their role in early stages of infection is not well understood (35). Th17 cells demonstrate significant plasticity, and during respiratory infections, Th17 cells can enhance early IFN-γ expression and acquire functional characteristics of Th1 cells (36, 37). In studies of pulmonary and intestinal mucosal immune responses, Th17 cells have been observed to differentiate into TRMs, to promote IgA translocation into the airways and differentiate into T-follicular helper cells that induce IgA class switching (38–40). Thus, vaccine-induced Th17 cells in the URT could have a beneficial role in early stages of infection with SARS-CoV-2 (36). We also observed the presence of TRMs in the nasal passages of URT-immunized mice, and following SARS-CoV-2 challenge, vaccination with Pam_2_Cys/spike protected mice from disease and lung pathology (**Figure 7**). We did not observe any immunopathology induced by nasal Pam_2_Cys/spike immunization, indicating the CD4^+^ T cells induced by this vaccine were not immunopathogenic. Future studies are needed to delineate the contribution of Th17 cells to the protection observed and their potential role in promoting IgA responses.

We did not observe induction of a significant population of antigen-specific CD8^+^ T cells, which are known to assist in providing antibody-independent protection from SARS-CoV-2, following either URT/LRT or URT delivery of Pam_2_Cys/spike (41). The incorporation of additional adjuvants may serve to broaden the T-cell repertoire induced. However, while CD8^+^ T cells contribute to protection against severe disease, CD4^+^ T cells are essential for the maintenance of long-term NAb responses and are associated with protection against milder disease (42, 43). Therefore although the Pam2Cys/spike vaccine did not activate CD8^+^ T cells, the vaccine generated cellular and humoral immunity sufficient to protect K18-hACE2 mice against viral challenge, confirming the protective roles of CD4^+^ T cells and antibodies against SARS-CoV-2.

Since the COVID-19 pandemic, multiple inhaled vaccines have been approved for use (4). iNCOVACC, an adenovirus-vectored IN vaccine produced by Bharat Biotech (India), was found to induce salivary IgA, promote higher NAb and cause fewer side effects in human participants than the intramuscular COVAXIN in a phase 3 clinical trial (44). In general, the intramuscular SARS-CoV-2 vaccines in clinical use are poor activators of mucosal immune responses, and antigen recognition in the respiratory mucosa appears to be required for generation of local immune memory (32, 33, 45–47). Any nasal vaccines that enter clinical use will act as booster vaccines to already approved intramuscular vaccines and/or be deployed in people with prior SARS-CoV-2 infection. The concept of prime-pull vaccination proposes that parenteral immunization followed by a mucosal ‘pull’ boost will recruit immune cells into the respiratory mucosa (24, 48). We showed in the current study that URT boosting of mice that have been previously immunized subcutaneously generates robust mucosal NAb and spike-specific Th17 cells (**Figures 5**, **6**). Furthermore, both a full schedule of mucosal vaccinations or mucosal boosting provided robust protection against delta SARS-CoV-2 infection (**Figure 7**). Though we did not compare URT boosting to parenteral (subcutaneous) boosting in this study, in previous studies, we and others have shown that mucosal compared to parenteral delivery promotes significantly greater mucosal immunity. Mao et al. demonstrated boosting mRNA-vaccinated animals intranasally with spike protein generates pulmonary humoral and cellular immune responses, suggesting similar results could be observed with URT boosting with Pam_2_Cys/spike (10, 24, 33). A limitation of this study is that URT Pam_2_Cys/spike vaccine was not tested as a booster after immunization with current clinically used COVID-19 vaccines, such as Spikevax (Moderna) or Comirnaty (Pfizer/BioNTech). Future studies should address if the ‘prime-pull’ phenomenon observed in the present study is maintained after mRNA vaccination. This comparison will be important to perform in future validation of URT vaccination with Pam_2_Cys/spike. In total, the data herein demonstrate URT Pam_2_Cys is an effective booster vaccine for inducing mucosal immune memory after prior parenteral priming.

This study only examined protection against homologous infection with delta SARS-CoV-2. Future studies will examine cross protection against more recent variants of concern, such as the JN-1 or FLiRT Omicron variants. We did observe low levels of cross-neutralizing antibodies against pseudovirus expressing BA.5 omicron spike protein, consistent with the Pam_2_Cys/D-spike vaccine providing some cross-protection. Finally, mouse models, such as those used in this study, are unable to test prevention of transmission, an important characteristic for the assessment of mucosal COVID-19 vaccine candidates. Future experiments should test the efficacy of this vaccine preventing viral transmission after vaccination using Syrian hamster transmission models (2, 49). In this study we have identified sterilizing protection by URT Pam2Cys/spike at 6 days post-infection, but early assessment of viral load in the nasal turbinates and nasal wash of mice, 1–2 days post-infection, could provide an indication that URT Pam_2_Cys/spike vaccination also protects from initial infection.

In summary, URT immunization with Pam_2_Cys/spike vaccines generated significant antigen-specific humoral and cell-mediated immune responses in the nasal passages and blood of mice, despite the low volume of vaccine restricting delivery to the upper airways (9). As a booster after parenteral vaccination, URT Pam_2_Cys/D-spike induced sterilizing protective immunity in the lungs and brain against delta SARS-CoV-2 challenge. These data support the clinical development of Pam_2_Cys-adjuvanted protein vaccines for nasal delivery.

## Materials and methods

### Vaccine adjuvant synthesis

The vaccine adjuvant Pam_2_Cys-SK_4_-triethylene glycolate (Cys-Dipalmitoyl-Ser-Lys-Lys-Lys-Lys-PEG_2_-NH_2_; Pam_2_Cys) was produced as previously described and detailed herein (10). Synthesis proceeded with the loading of Fmoc-Peg_2_-OH (166108-71-0) to rink amide resin. First, the Rink Amide resin was treated with piperidine in DMF (1:4, v/v) to remove the Fmoc group, then the resin was washed with DMF, CH_2_Cl_2_ and DMF again. The resin was then treated with a solution of Fmoc-Peg_2_-OH, Oxyma and *N*,*N’*-Diisopropylcarbodiimide in DMF. The remaining Ser-Lys-Lys-Lys-Lys portion of the peptide was extended in the same manner, using Fmoc-Lys(Boc)-OH and Fmoc-Ser(*t*Bu)-OH. The Pam_2_Cys unit was installed using a Fmoc-S-[(R)-2,3-bis(palmitoyloxy)propyl]-L-cysteine building block. The N-terminal Fmoc group was then liberated with piperidine in DMF (1:4, v/v), and the resin was washed with DMF then CH_2_Cl_2_. The peptide was then liberated from resin and its sidechain protecting groups with an acidolytic treatment with TFA/*i*-Pr_3_SiH/H_2_O (90:5:5, v/v/v). The crude residue was purified by reverse-phase HPLC, with the buffers of water (0.1% formic acid) and acetonitrile (0.1% formic acid). The HCl salt of Pam_2_Cys-SK_4_-Peg_2_-NH_2_ was formed through iterative freeze-drying following re-solubilization in dilute aqueous HCl.

### Mice and immunizations

Female C57BL/6 mice 6–8 weeks of age were purchased from Animal BioResources (Moss Vale, Australia) and housed at the Centenary Institute under specific pathogen-free conditions. As all experiments were performed using female mice, sex was not accounted for as a biological variable in this study. SARS-CoV-2 HexaPro ancestral spike protein was expressed in Expi293F cells using methods described previously (10). Delta-variant SARS-CoV-2 HexaPro spike protein was obtained from Excellgene (Monthey, Switzerland). Mice were randomly allocated to an experimental group and immunized with 5 µg Pam_2_Cys and 6 µg spike protein (ancestral or delta variant) for all experiments except where otherwise stated in the figure legend. All immunizations were performed while mice were anaesthetized under inhalational isofluorane (1 L/min O_2_ and 4% isofluorane). Subcutaneous injections were performed in a total volume of 200 µL Dulbecco’s phosphate buffered saline (PBS; Sigma Aldrich, MA, USA) in the back skin, URT/LRT immunizations were performed using a total volume of 30 µL, and URT immunizations in a total volume of 12 µL to the nares (6 µl each nostril).

To validate URT immunizations were not being delivered to the lungs, a pilot experiment was performed where C57BL/6 background female mice were injected intraperitoneally with 200 µL ketamine (mg/kg) and xylazine (10 mg/kg) for anesthesia. Once fully sedated, 6 µL trypan blue dye (Gibco, Life Technologies, USA) was administered using a pipette to each nare. Mice were left sedated to inhale the dye for 5 minutes before being culled using CO_2_. The lungs, stomach, nasal turbinates, trachea and esophagus were then examined for trypan blue staining (**Supplementary Figure S3**). None of the mice that received IN trypan blue displayed any evidence of inhalation to the lungs, but trypan blue was clearly visible in the nasal turbinates of mice. There was also evidence of trypan blue on the tongue and in the esophagus, but none in the stomach.

### SARS-CoV-2 challenge

Hemizygous female K18-hACE2 mice that express the human angiotensin converting enzyme 2 (hACE2) were immunized with Pam_2_Cys and D-spike protein either twice SC followed by URT or a full schedule of URT delivery (**Figure 7A**). SC vaccinated mice were first immunized with 5 µg Pam_2_Cys and 6 µg D-spike protein, followed by a booster with 3 µg Pam_2_Cys and 6 µg D-spike protein. All URT vaccinations were performed with the same dose of 5 µg Pam_2_Cys/6 µg D-spike protein. Eight weeks after immunization mice were challenged with delta-variant SARS-CoV-2 as described previously (33, 50, 51). Briefly, mice were anaesthetized using isoflurane prior to intranasal challenge with SARS-CoV-2 (1,000 PFU, delta variant B.1.617.2) in a 30 µL total volume of PBS. Mice were weighed and monitored for clinical symptoms daily as previously described (33, 50, 51) and were euthanized at day 6 post-infection. Viral loads were determined in the BALF, lungs, and brain by plaque assay. Total leukocytes in the BALF were enumerated with a total cell count using a hemocytometer, after which cells were spun onto glass slides using a cytospin, all as previously described (50). Slides were then stained using a Quick Dip Stain Kit (Modified Giemsa Stain) protocol as per the manufacturer’s instructions (POCD Scientific, Australia) and differential cell counts obtained. Lung histology on formalin-fixed paraffin-embedded blocks was performed as previously described (33, 50–52).

### Collection of tissue samples

During some experiments, blood was collected by tail vein for serum. At the endpoint, mice were euthanized by asphyxiation with CO_2_, before samples (blood, nasal wash, BALF, lungs, cervical lymph nodes, nasal turbinates and NALT) were collected aseptically. Blood was obtained via the inferior vena cava/portal vein, allowed to clot, then centrifuged (2000 xg, 15 min) for collection of sera. The BALF was collected via tracheal intubation, inflation of the airways with 1 mL DPBS and collection of the resultant fluid. Any cells were then removed by centrifugation, and the BALF was immediately frozen at -30°C. Nasal washes were collected by flushing 300 µL DPBS through the nares via the trachea while the mouse was laid supine. To collect lung tissue, any circulating blood was removed by perfusion with PBS and heparin (20 U/mL, Sigma) injected into the right atrium of the heart. For some experiments, the apical lobe was inflated with 10% neutral buffered formalin and stored at room temperature for future histological analysis. The remaining lung lobes were diced and then digested (45 min, 37 °C) with collagenase type 4 (50 U/mL, Sigma) and DNase I (13 μg/mL, Sigma), before being filtered through 70 μm sieves. Nasal turbinates were similarly digested and filtered through 70 µm sieves. Cervical lymph nodes and NALT were passed through 70 μm filters, and the cells pelleted by centrifugation. Lysis of erythrocytes was performed using ACK lysis buffer (Gibco, Thermo Fisher Scientific, MA, USA) where necessary, and then leukocytes were enumerated using a Countess 3FL (Invitrogen, MA, USA) with Trypan Blue (0.4%; Invitrogen) exclusion.

To determine antigen-specific T cell responses, cells were incubated in the presence of 5 µg/mL spike protein (either delta or ancestral variant depending on the experiment) and spike peptide 538–546 at 5 µg/mL concentration (epitope identified by Zhuang et al., to be recognized by CD8^+^ T cells (53)) for 4 hours at 37°C, before the addition of 10 µg/mL Brefeldin A (Sigma) and incubation overnight at 37°C.

### Staining of single cell suspensions for flow cytometry

Single cell suspensions were added to a 96-well round bottom plate for staining. Cells were first stained for viability by incubation at 4°C with Live/Dead stain blue (Invitrogen) and Fc block (clone 2.4G2, Becton Dickinson (BD) NJ, USA, catalogue number 553142) in PBS. Surface staining was achieved by incubation of antibodies (**Supplementary Table S1**) with cells (4°C in 2% fetal calf serum (FCS), 2 mM EDTA in PBS). For intracellular cytokine staining, cells were then permeabilized and fixed using the BD Cytofix/Cytoperm Fixation/Permeabilization Kit followed by incubation with fluorescently conjugated monoclonal antibodies. For intracellular transcription factor staining, cells were permeabilized and fixed using the eBioscience Foxp3/Transcription Factor Staining Buffer Set (ThermoFisher Scientific) followed by incubation with monoclonal antibodies. Samples were analyzed on a BD LSR-II cytometer at the Sydney Cytometry Facility (Charles Perkins Centre, NSW, Australia). Flow cytometric data was analyzed using the gating strategies shown in **Supplementary Figure S4**.

### Assessment of humoral immune responses

Spike-specific antibody titers were determined by coating Corning 96-well Clear Flat Bottom Polystyrene High Bind Microplates (Corning, NY, USA) with 1 µg/mL spike protein (ancestral or delta) in sodium carbonate coating buffer (0.05M pH 9.6; 1.59 g/L Na_2_CO_3_, 2.93 g/L NaHCO_3_), 70 µL per well, then incubating at 4°C overnight. Plates were then washed with PBS (POCD) and 0.05% Tween 20 (Sigma), before being blocked with 1% bovine serum albumin (BSA; Bovogen, Vic, Australia) in PBS for (1 hour, 37°C). Serum samples were serially diluted in 1% BSA/PBS then added to washed microplates and incubated (1 hour, 37°C). Secondary antibodies conjugated to HRP were then added diluted 1 in 2000 in 1% BSA/PBS and incubated (30 minutes, 37°C). Antibodies used were anti-mouse IgG is Novex (#A16090, Life Technologies, CA, USA), anti-mouse IgG1 (#115-035-205, Jackson ImmunoResearch, PA, USA), anti-mouse IgG2c Jackson (#115-035-208, ImmunoResearch), and anti-mouse IgA (#626720, Invitrogen). Alternatively, antigen-specific IgA was detected using biotinylated anti-mouse IgA (#ab97233, Abcam), followed by washing and SA-HRP (#21130, Thermo Scientific Pierce High Sensitivity). Plates were then washed and TMB substrate (Sigma) was added and allowed to develop for approximately 2 minutes before the reaction was stopped by the addition of an equal volume of 2M HCl (Sigma). Absorbances were read at 450 nm (570 nm reference) on a Tecan plate reader.

### Pseudovirus assays

Pseudovirus assays were performed as described previously (33). Briefly, HEK293T cells transduced to express human ACE-2 were added to a 384-well plate (poly-D-lysine coated; PerkinElmer, MA, USA) and incubated at 37°C. The next day, serially diluted serum, BALF or nasal wash from immunized animals was incubated with pseudo-viruses expressing spike protein from ancestral, delta or omicron BA4/5 strains of SARS-CoV2. The pseudo-viruses were then added to ACE2-HEK293T cells, and the levels of infection were measured by fluorescence using a Phenix high throughput microscope (Sydney Cytometry Facility). The proportion of infected cells was enumerated using Harmony^®^ high-content analysis software (Perkin Elmer). NAb titers were determined as the dilution required for ≥50% inhibition of infection (EC50) compared to the infection levels of adjuvant-only controls, estimated by sigmoidal curve and interpolation (GraphPad Prism 10).

### Statistical analysis

Statistical analysis of differences between groups was performed using GraphPad Prism^®^ (GraphPad Software Inc., CA, USA), using the tests specified in the figure legends.

## Data Availability

The raw data supporting the conclusions of this article will be made available by the authors, without undue reservation.
