# Supplementary material for: Upper respiratory tract immunization with Pam2Cys-adjuvanted spike protein vaccine achieves sterilizing protection against SARS-CoV-2
Source: Front Immunol. 2025 Sep 2;16:1654126. doi: 10.3389/fimmu.2025.1654126 (PMC12436419; doi:10.3389/fimmu.2025.1654126)
Supplement: Supplementary file 1 [file DataSheet1.pdf]

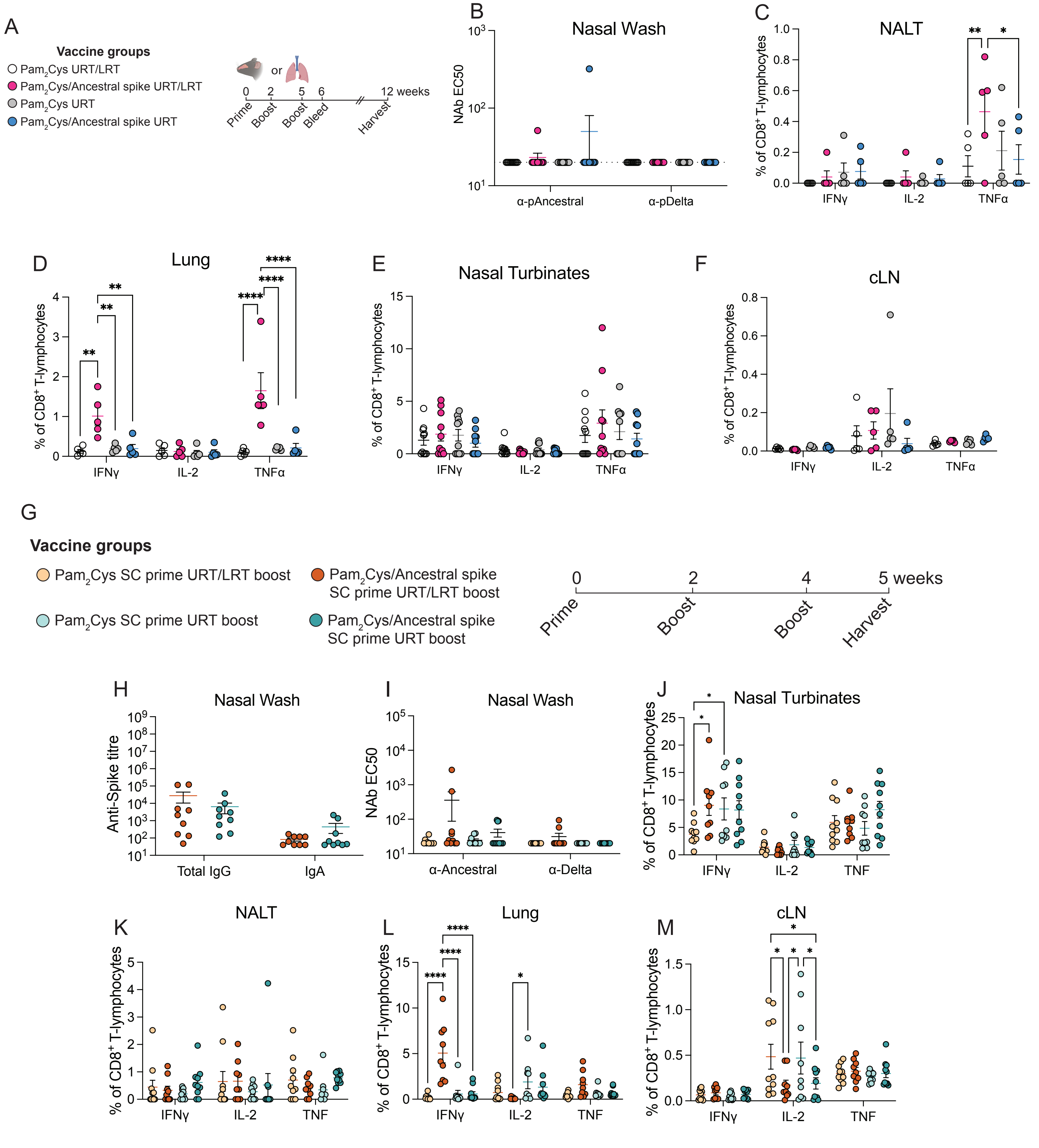

Supplementary Figure 1.
Mice were immunized with 5 µg Pam2Cys and 6 µg ancestral spike protein via the schedules outlined in (A) and (G). Tissues were collected either 1 week or 8 weeks post-immunization. For (B), nasal wash was measured for presence of neutralizing antibodies (NAb) using pseudovirus assay. (C-D) Tissues were examined for presence of antigen-specific CD8+ T cells by restimulation with anestral spike protein eight weeks after the final immunisation. (H) Total spike-binding antibodies in the nasal wash were measured by ELISA. (I) Nasal wash was examined for the presence of NAb using pseeudovirus assay. (J-M) Tissues were examined for presence of antigen-specific CD8+ T cells by restimulation with ancestral spike protein one week after the final immunisation. Differences between groups were analysed using a 2-way ANOVA with post-hoc Tukey test, except for in (H) which were analysed using a two-tailed Mann-Whitney test. For all graphs $p < 0.0332$(*), $p < 0.0021$(**), $p < 0.0002$ (***), $p < 0.0001$(****).

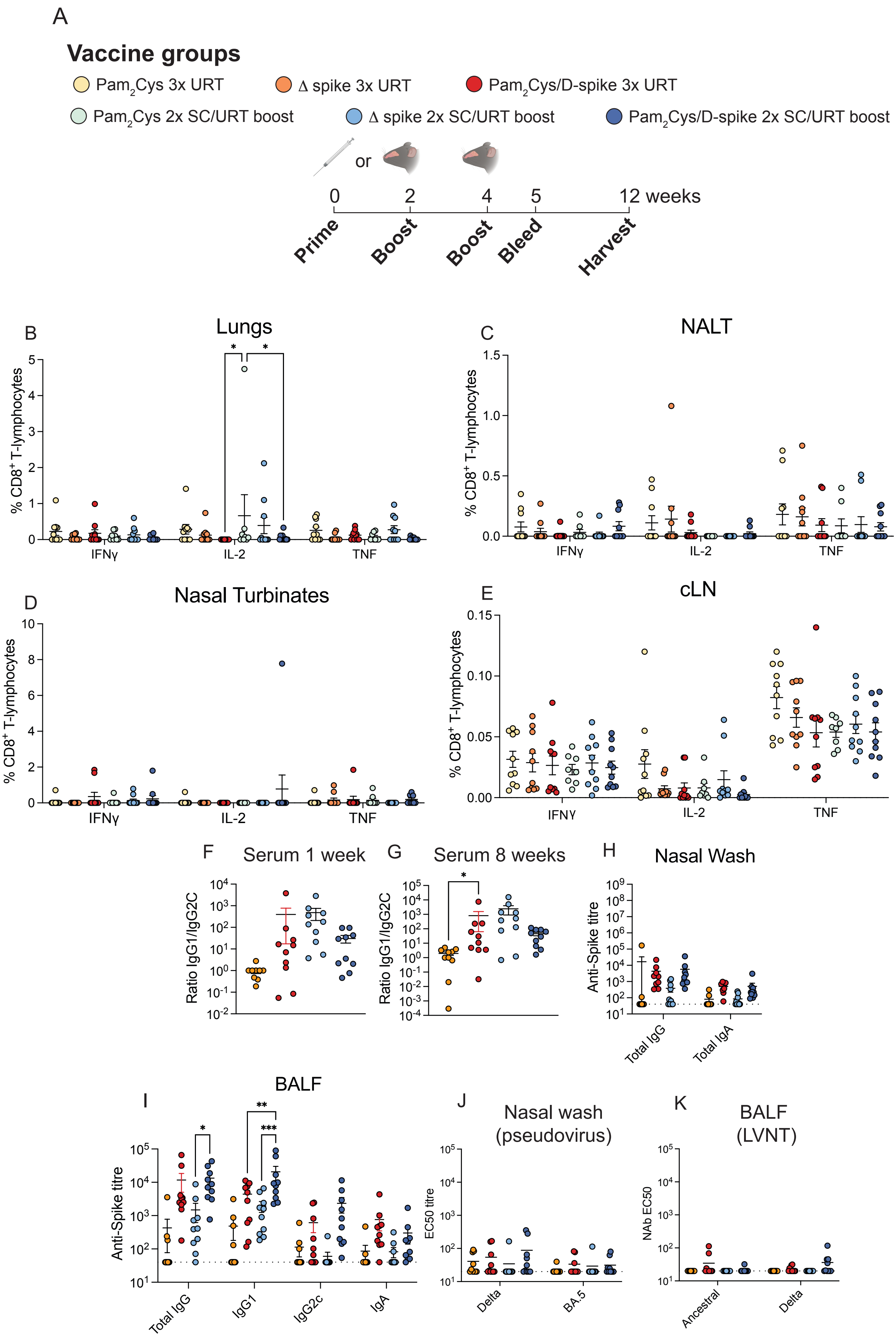

Supplementary Figure 2.
Mice were immunized with 5 µg Pam2Cys and 6 µg delta spike protein via the schedule outlined in (A). Samples were collected 8 weeks post-immunization. Tissues were examined for presence of antigen-specific $CD8^+$ T cells by restimulation with delta spike protein eight weeks after the final immunisation (B-E). For (F-I), spike-specific antibodies were identified via ELISA. For (J, K), NAb were identified using either pseudovirus (J) or live virus neutralisation (K) assays. Differences between groups were analysed using two-tailed Mann-Whitney test, or 2-way ANOVA with post-hoc Tukey test. For all graphs $p < 0.0332$(*), $p < 0.0021$(**), $p < 0.0002$ (***), $p < 0.0001$(****).

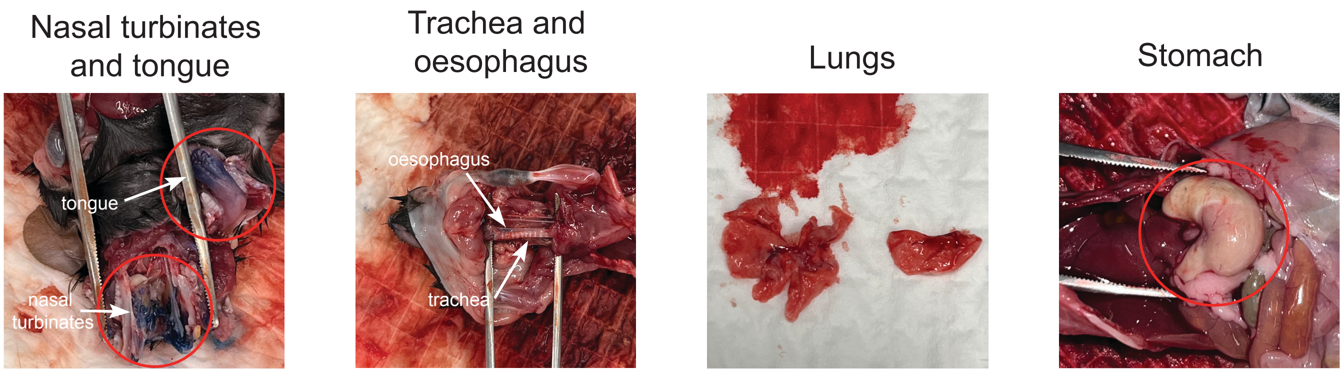

Supplementary Figure 3: Distribution of trypan blue after URT delivery.
Female C57BL/6 mice (n=6) were anaesthetized and administered 6 μL per nare of trypan blue. Representative images are shown of trypan blue distribution in tissues 5 minutes after intranasal delivery.

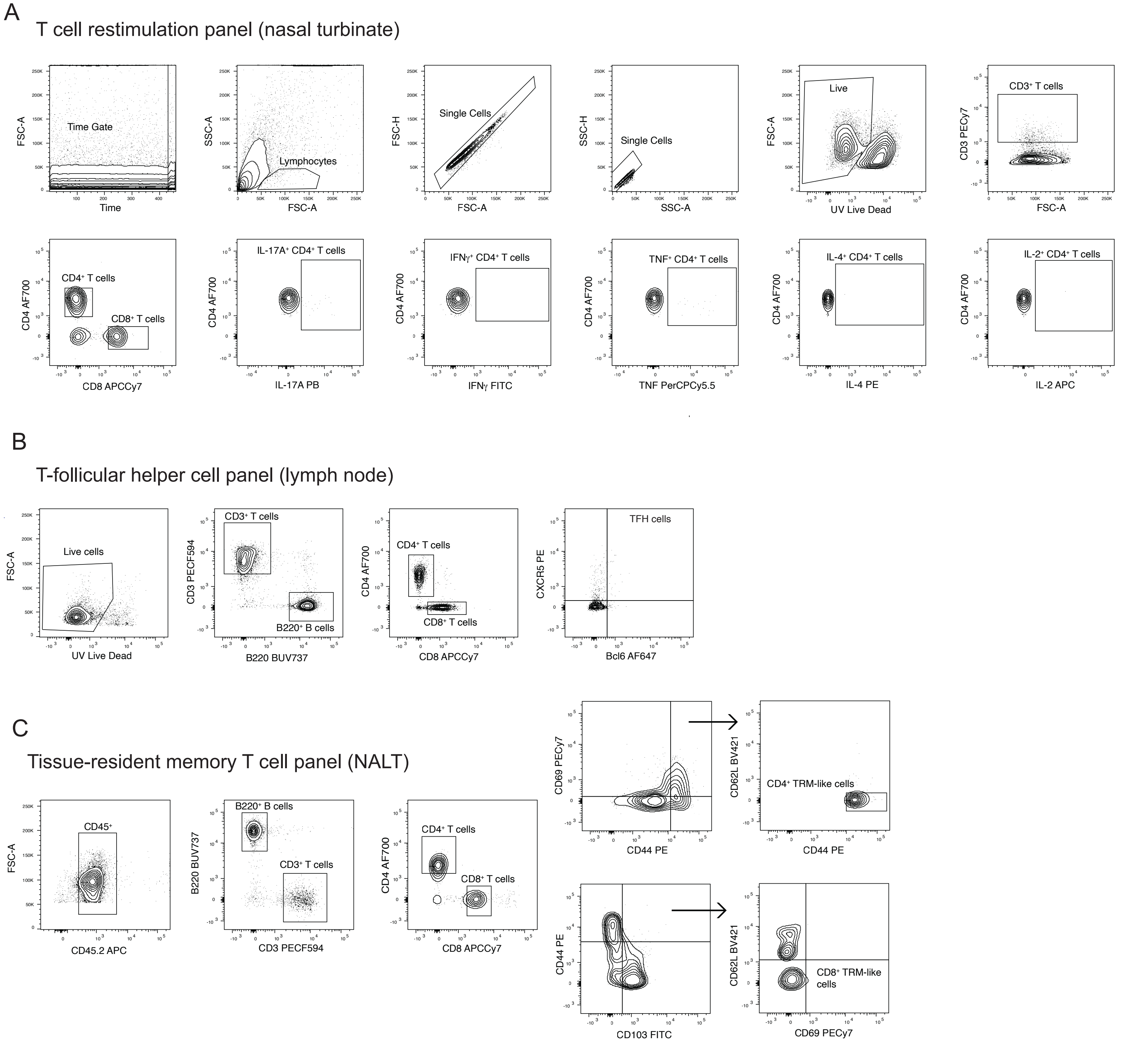

Supplementary Figure 4: Flow cytometry gating strategies used in this study.
Restimulated single cell suspensions were analysed for cytokine expression using the panel depicted in (A). Cells were gated on time, lymphocytes, single cells as per (A) then T-follicular helper cells in the lymph node were analysed as shown in (B). Cells were gated on time, lymphocytes, single cells, and live cells as per (A, B) then tissue-resident memory T cell (TRM)-like cells were gated as shown in (C).

Supplementary Table 1: Antibodies used in this study.

Antibody	Company	Catalogue	Lot	Clone
CD8 APCCy7	Becton Dickinson (BD)	557654	8319924	53-6.7
CD4 AF700	Biolegend	100536	B248742	RM4-5
CD3 PECy7	BD	100319	B282896	145-2C11
IFNy FITC	BD	554411	9197282	XMG1.2
IL-17A PB	Biolegend	506918	B230225	TC11-18H10.1
IL-4 PE	BD	554435	9319911	11B11
TNFa PerCPCy5.5	BD	560659	0227403	MP6-XT22
IL-2 APC	Biolegend	503810	B281632	JES6-5H4
CXCR5-Biotin	BD	551960	0160485	2G8
B220 BUV737	Life Technologies	L23105	1784607	
PD1 BV786	Biolegend	135225	B337077	29F.1A12
CD3 PECF594	BD	562286	0066150	145-2C11
Streptavidin-PE	BD	554061	0000034110	
CD44 PECY7	BD	560569	0100100	IM7
BCL6 AF647	BD	561525	1067317	K112-91
CD62L BV421	BD	562910	9189629	MEL-14
CD103 FITC	Biolegend	121419	B274032	2E7
CD44 PE	Biolegend	103008	B334370	IM7
CD69 PECY7	BD	552879	9098844	H1.2F3
CD45.2 APC	BD	558702	5092510	104
